# ABCD of Safe Dental Practice

**DOI:** 10.5005/jp-journals-10005-1079

**Published:** 2011-04-15

**Authors:** K Sunil Babu, V Thimma Reddy B, C Pujita Reddy, Sree Lalita

**Affiliations:** 1 Senior Lecturer, Department of Pedodontics and Preventive Dentistry, Mamata Dental College Khammam, Andhra Pradesh, India; 2 Professor and Head, Department of Pedodontics and Preventive Dentistry, Mamata Dental College Khammam, Andhra Pradesh, India; 3 Senior Lecturer, Department of Oral and Maxillofacial Surgery, MNR Dental College, Sangareddy, Andhra Pradesh, India

**Keywords:** Practice management, Professionalizm, Ethical practice.

## Abstract

Dental practice is the integral component of the oral health. Though the dental practice is in close relation with that of the medical practice, it has its own distinctiveness in relation to safe practice. The safe dental practice should not only assure good oral and general health but also improve social interaction by enhancing physical appearance, esthetics, etc. For the safe dental practice, dentists must excel in patient care and standard of treatment. The interlocking missions of education, research, and patient care are the cornerstones for the safe and healthy dental practice. This paper is designed to bridge the gap between the educational preparation of the dentist and the reality of the working world in a simple way.

## INTRODUCTION

It is a common belief among many individuals that being ‘good with people’ is an inborn art and owes little to science or training. It is true that some individuals have a more open disposition and can relate well to others. However, there is no logical reason why all of us should not be able to put patients at their ease and show that we are interested in their problems. It is particularly important for dentists to learn how to help people relax, as failure to empathize and communicate will result in disappointed patients and an unsuccessful practicing career. Communicating effectively with children is of great value, as ‘being good with patients’ is a practice-builder and can reduce the stress involved when offering clinical care.

As dentists, we cannot be considered competent without good practical clinical skills, but evidence shows that patients judge our technical competence on many factors. One among them is communication, which plays an important role in building up our clinical practice. According to Professor Liz Kay, Dean of the Peninsular Dental School says: ‘It is not possible to truly “help” and “care” for patients in a way that will cause them to trust you without good interpersonal skills. It is by your communication with your patients that they judge you’.^1^

## KEYS TO SUCCESSFUL MANAGEMENT

The safe dental practice in this article for the practitioners are explained in a simple ABCD format that are expanded below in detail:

### A

- Ability of the dentist to communicate.
- ‘Acceptance Universally’^2^ is as important as universal health care. When the dentist makes the sacrifice to provide oral health care based on need rather than for payment, he or she affirms the worth of each patient as a fellow human who has the right to have basic needs met. Adequate training to the staff, particularly the patient management and communication skills.^3^
- Advertisement does not need to be brash, after all, it is merely a means of letting the public know about the existence of a practice and the services available.
- Alertness of dentist
- Allow only one attender with a patient
- Appearance of dentist
- Always available for patients
- Always listen to the patient first
- Anxious patient: Long periods of time in the waiting room can become very stressful. A dental office that repeatedly makes a patient wait more than 20 minutes sends a signal to the patient that their time is not respected.^4^
- Avoid cross infection.

### B

- Behavior of the dentist towards the patient: Understanding the fundamentals of human behavior is a critical competency for practitioners.^5^
- Being completely reliable and ethical to both patients and codentists.
- Be legal, decent, honest, and truthful
- Be patient to get more patients
- Body language of the dentist.

### C

- Case sheet updating and maintaining it properly
- Cell phone consultation—no
- Clients (patients and relatives) satisfaction—a priority
- Clinical audit is the systematic critical analysis of the quality of dental care covering procedures used for diagnosis and treatment, use of resources, and the outcome of the patient. The aim is to encourage dentists to self-assess different aspects of their practice, implement changes, and monitor them with a view to improve service and patient care.^3^
- Colleague help—second and third opinion particularly for any special and systemically involved cases.
- Coffee breaks and lunch time should not always be used to catch upon other work; be kind to yourself and have rest occasionally.^3^
- Comment loosely on other dentist—never
- Communicate openly in oral or written without concealing important relevant facts
- Complaints of patients should be attended immediately
- Condition should be explained at the time of first dental visit, if diagnosed
- Confidence of patient and attendant should be gained
- Confirm clinical diagnosis by relevant investigations
- Consent on the case sheet (procedure particularly in surgical case)
- Consistent adherence to standards and values toward patients and codentists
- Consumer protection act and its implications should be known to every dentist
- Convince clearly and confidently
- Cool, calm and composed
- Core knowledge and clinical accumen update by continuous daily reading, attending CDE’s, conferences, and clinical meetings regularly.
- Cost of treatment to be explained in advance
- Cross reference to specialist as and when necessary.

### D

- Debt management:^6^ Properly structuring debt will allow you to acquire the things you need and want now, and at the same time, not neglect saving for the future. Compounding those savings truly is magic.
- Declaration of any mistake in the procedure—it is an art.
- Decrease distraction (cell phone, cricket, computer cinema and serials, etc).
- Degree of dentist should be valid with DCI registration
- Delegate those tasks that do not require your training, reduces stress, and increase job satisfaction to ancillary staff.^3^
- Disposal of waste
- Document on case sheet—detailed history/examination findings/investigations/diagnosis and treatment required.
- Dues collection from patient taken promptly.

If we follow above mentioned ABCD, we will get:

D-Dollar (money)

E-Enjoy life

F-Famous dentist

G-Gratification

H-Happiness always in the long run.

If we do not follow these simple steps of ABCD, it may lead to:

- Damage to the dental practice
- Defamation to the dental clinic
- Depression of the dentist which may further lead to frustration and loss of hope
- Disappointment of the patient.

## CONCLUSION

In this competitive world of dental practice, one should maintain standards in terms of updating knowledge, patience, ethics, communication, etc. for a safe dental practice. A happy practice environment is not only more pleasant to work in, but the bonhomie will also be transmitted to the patients. Thus, it is very important to follow the above mentioned ABCD for tension and stress free practice, with professional satisfaction, more prosperity, happy and peaceful family life.
